# Application of InceptionV3, SqueezeNet, and VGG16 Convoluted Neural Networks in the Image Classification of Oral Squamous Cell Carcinoma: A Cross-Sectional Study

**DOI:** 10.7759/cureus.49108

**Published:** 2023-11-20

**Authors:** Deepak Pandiar, Sahil Choudhari, Reshma Poothakulath Krishnan

**Affiliations:** 1 Oral Pathology and Microbiology, Saveetha Dental College and Hospitals, Saveetha Institute of Medical and Technical Sciences, Saveetha University, Chennai, IND; 2 Conservative Dentistry and Endodontics, Saveetha Dental College and Hospitals, Saveetha Institute of Medical and Technical Sciences, Saveetha University, Chennai, IND

**Keywords:** oscc, oral squamous cell carcinoma, neural network, image analysis, artificial intelligence

## Abstract

Background

Artificial intelligence (AI) is a rapidly emerging field in medicine and has applications in diagnostics, therapeutics, and prognostication in various malignancies. The present study was conducted to analyze and compare the accuracy of three deep learning neural networks for oral squamous cell carcinoma (OSCC) images.

Materials and methods

Three hundred and twenty-five cases of OSCC were included and graded histologically by two grading systems. The images were then analyzed using the Orange data mining tool. Three neural networks, viz., InceptionV3, SqueezeNet, and VGG16, were used for further analysis and classification. Positive predictive value, negative predictive value, specificity, sensitivity, area under curve (AUC), and accuracy were estimated for each neural network.

Results

Histological grading by Bryne's yielded significantly stronger inter-observer agreement. The highest accuracy was found for the classification of poorly differentiated squamous cell carcinoma images irrespective of the network used. Other values were variegated.

Conclusion

AI could serve as an adjunct for improvement in theragnostics. Further research is required to achieve the modification of mining tools for greater predictive values, sensitivity, specificity, AUC, accuracy, and security. Bryne's grading system is warranted for the better application of AI in OSCC image analytics.

## Introduction

Oral squamous cell carcinoma (OSCC) is a lethal malignancy with high mortality and morbidity worldwide [1,2]. The survivors face grave outcomes in terms of function, aesthetics, and overall quality of life, mandating the early diagnosis and treatment of OSCC. This is further complicated by non-specific clinical presentations. Non-habit-related and biological agents in the pathogenesis of OSCC are other well-established challenges. Histopathological analysis of biopsied tissue forms the backbone of management and prognosis. Histologically, OSCC is graded in a three-tier or four-tier grading system. Many pathologists prefer the grading system put forwarded by the World Health Organization (WHO), which relies upon the resemblance of tumor cells to normal squamous epithelium and the amount of keratin produced. Unfortunately, this grading system bears a weak correlation with disease outcome and survival [3]. Tumor grading at deep invasive front, which not only considers the degree of keratinization but also includes pattern of invasion, intensity of inflammatory infiltrate, and nuclear pleomorphism, bears high prognostic value [3,4]. Further, this grading system yields good inter-observer agreement among pathologists.

Previous studies have demonstrated the role of deep learning (DL) in the prognostication of malignancies of the lungs, breast, colon, bladder, and many more [5-9]. It has also been established that artificial intelligence (AI) works at par with conventional microscopy for primary diagnosis in surgical pathology [10]. Briefly, DL, a part of the wide field of AI, may be divided into two DL-based unsupervised feature learning and handcrafted approach [11]. With the application of convoluted neural network (CNN), in the former, segmentation of images, object detection, and classification have been attempted for OSCC [12]. Various CNNs are available for the task such as InceptionV3, U-Net, ResNet-50, and AlexNet, to name a few [11]. Previous studies attempted the application and comparison of these CNNs for the classification of OSCC images. The results of DL in surgical pathology are promising and provide multiple advantages, such as the detection and quantification of morphological features, and thus may act as a decision support system in routine pathology practice. Still, the data pertaining to the comparison of these unsupervised neural networks with conventional microscopy is meager, and much remains to fill the gap between manual methods of quantification and unsupervised/handcrafted DL methods. The present study was thus orchestrated for the comparison among three CNNs using the Orange data mining tool. Further, the objectives of the study were to assess the inter-observer agreement for Bryne's histopathological grading system of OSCC and detailed analysis of positive predictive value (PPV), negative predictive value (NPV), sensitivity, specificity, area under curve (AUC) for linear regression, and accuracy for all the three selected neural networks.

## Materials and methods

**Figure 1:**
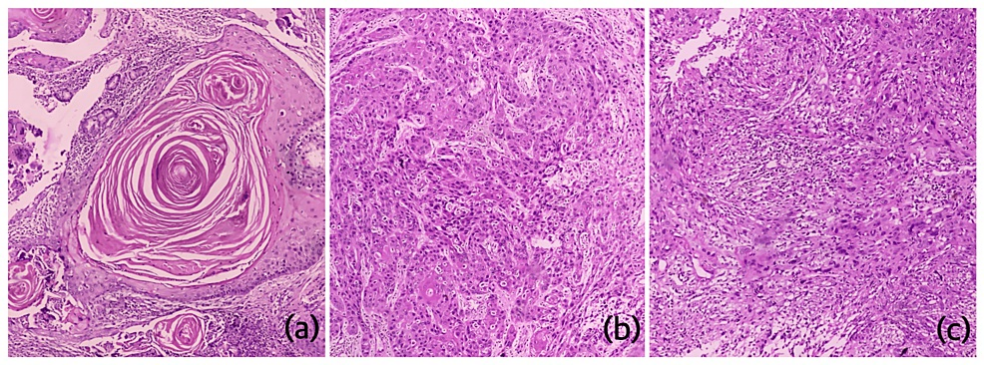
Photomicrographs of H&E-stained sections showing well-differentiated squamous cell carcinoma (a), moderately differentiated squamous cell carcinoma (b), and poorly differentiated squamous cell carcinoma (c) (100X) H&E: hematoxylin and eosin

The present cross-sectional comparative study included 325 consecutive cases of OSCC reported to the Department of Oral Pathology and Microbiology of Saveetha Dental College and Hospitals, Chennai, India, irrespective of the nature of the biopsy (incisional or excisional), and was conducted from April to June 2023. As the study included retrieval of histopathological images from archives of an online data mining tool, ethical clearance was not required. Uniformity in the color and shade was maintained, as all the slides were stained by a single expert technician using departmental standard operating protocol. All the slides were evaluated by two expert oral pathologists (DP and RPK). Both the observers independently graded the cases into well-differentiated squamous cell carcinoma (WDSCC), moderately differentiated squamous cell carcinoma (MDSCC), and poorly differentiated squamous cell carcinoma (PDSCC) using the WHO grading system and Bryne's grading system (Figure 1). Bryne's grading system yields high inter-observer agreement and bears high prognostic value unlike Broder's grading system. Hence, Bryne's grading system was chosen over Broder's classification. 

The latter system semi-quantitatively analyzes the tumor invasive pattern, keratinization, nuclear pleomorphism, and degree of lymphoplasmacytic infiltrate in a four-point system at the invasive tumor front (ITF) [4]. Inter-observer variability was determined by kappa statistics.

Thereafter, the Orange data mining tool (https://orangedatamining.com) was used for further analysis, classification, and assessment [13]. Orange image analytics comprise components for loading the images, embed these images into a vector space, and analyze the image profiles for inferring image clusters and classification. Data analysis in Orange data mining is implemented through workflows; for image classification, the software provides an interface to various DL models such as InceptionV3, SqueezeNet, Painters, VGG16, VGG19, DeepLoc, and OpenFace. We used InceptionV3, SqueezeNet, and VGG16 for further analysis and classification. The software allows the evaluation of predictive value (PV), logistic regression, precision, and accuracy for classifiers.

Briefly, PVs were used to elucidate the outcome of the three neural networks by examining the correct classification of included images by the tests and were further subclassified into PPV and NPV. For estimating correlation and interrelationship between a dependent variable and one or more independent variables, logistic regression was employed. Precision and accuracy are not dependent on each other. While accuracy depicts the closeness of any measurement to the true or accepted value, precision estimates the closeness of the measurements of one item to each other.

## Results

Routine histopathology

Three hundred and twenty-five cases were independently graded by two widely used grading systems, viz., WHO grading system and Bryne's grading system. There were 142 images of WDSCC, 119 images of MDSCC, and 64 images of PDSCC, 325 cases in total. Among the two systems, kappa values of 0.475 and 0.835 were found, respectively, for WHO and Bryne's system, suggesting a weak inter-observer agreement for the WHO grading of OSCC. Inter-observer agreement for the latter system was strong. The results were statistically significant (p-value of <0.05). The major discrepancy was seen in grading moderately differentiated OSCC using WHO classification, which was overcome by adding more parameters, such as the pattern of invasion, lymphoplasmacytic infiltrate, and nuclear pleomorphism, in addition to keratinization. Although, in 1992, Bryne's grading mitoses were excluded, we found that inclusion of this parameter improved inter-observer agreement (kappa value of 0.925).

DL

**Figure 2:**
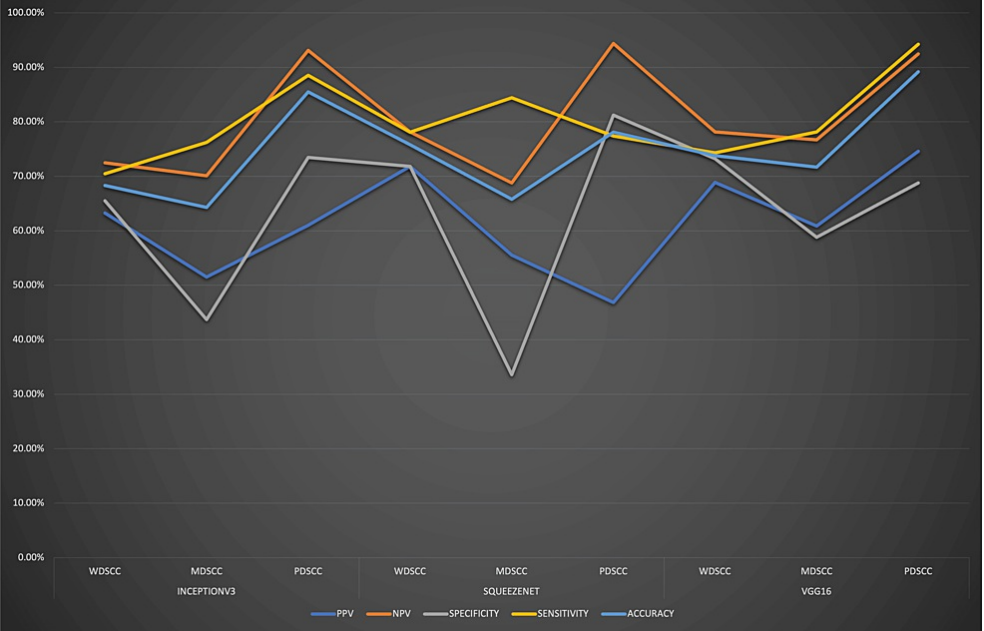
Pictorial presentation of performance comparison of the three convoluted neural networks used in detecting oral squamous cell carcinoma for PPV, NPV, specificity, sensitivity, and accuracy PPV: positive predictive value; NPV: negative predictive value; WDSCC: well-differentiated squamous cell carcinoma; MDSCC: moderately differentiated squamous cell carcinoma; PDSCC: poorly differentiated squamous cell carcinoma

Three DL models used in the present study were InceptionV3, SqueezeNet, and VGG16. InceptionV3, a DL tool, is based on ImageNet and showed an accuracy of 68.3%, 64.31%, and 85.5% for WDSCC, MDSCC, and PDSCC, respectively. SqueezeNet, a DL model for image recognition, has been proposed to achieve AlexNet-level accuracy on ImageNet and demonstrated an accuracy of 75.8%, 65.8%, and 78.1% for WDSCC, MDSCC, and PDSCC, respectively. Finally, VGG16, a 16-layered image recognition model trained on ImageNet, yielded 73.8%, 71.7%, and 89.2% accuracy, respectively, for WDSCC, MDSCC, and PDSCC. The detailed comparative values of PPV, NPV, sensitivity, specificity, AUC for linear regression, and accuracy for all three neural networks are shown in Table 1 and Figure 2.

**Table 1:** Detailed values using the three neural networks for all histological grades of oral squamous cell carcinoma PPV: positive predictive value; NPV: negative predictive value; AUC: area under curve; WDSCC: well-differentiated squamous cell carcinoma; MDSCC: moderately differentiated squamous cell carcinoma; PDSCC; poorly differentiated squamous cell carcinoma

	InceptionV3			SqueezeNet			VGG16		
	WDSCC	MDSCC	PDSCC	WDSCC	MDSCC	PDSCC	WDSCC	MDSCC	PDSCC
PPV	63.26%	51.5%	61.03%	71.83%	55.55%	46.85%	68.87%	60.87%	74.58%
NPV	72.47%	70.09%	93.14%	78.14%	68.77%	94.39%	78.16%	76.67%	92.48%
Specificity	65.49%	43.7%	73.44%	71.83%	33.61%	81.25%	73.24%	58.8%	68.8%
Sensitivity	70.49%	76.21%	88.51%	78.1%	84.4%	77.4%	74.32%	78.16%	94.25%
AUC	0.867	0.790	0.933	0.833	0.748	0.902	0.788	0.715	0.928
Accuracy	68.3%	64.31%	85.5%	75.8%	65.8%	78.1%	73.8%	71.7%	89.2%

## Discussion

Diagnosis and assessment of prognosis are the two main factors that need to be considered for any disease process, malignancies in particular. Concerning the malignant neoplasms of the head and neck region, oral cancer remains the most common malignancy globally, with approximately 354,864 new cases diagnosed annually. Out of these, OSCC constitutes for 90% of all the head and neck malignancies. With 377,713 new cases/year and 177,757 annual deaths, OSCC also ranks 16th among all the malignant neoplasms [14]. Despite magnificent advances in theragnostics, the morbidity and mortality of OSCC patients have not improved much [15,16]. Among the recent advances, DL, a part of AI, has shown some promising results in different studies pertaining to cancers of the lungs, oral cavity, breast, colon, and bladder [11]. Additionally, AI has been applied in immunofluorescence and immunohistochemistry image analysis [17]. Previous studies regarding the application of DL in image analysis, segmentation, and classification of OSCC images have yielded variable results. 

In general, AI exploits the identification of specific parameters and knowledge of neoplastic tissues and learns either, automatically (unsupervised) or by manual approach (handcrafted approach), with the selection of appropriate meaningful features resulting in maximum class separability. Therefore, the selection and application of an appropriate grading system is essential for the selection of images. Broder's or WHO grading systems lack any prognostic value, while in contrast, it has been reported that modified Bryne's system for grading OSCC predicts survival and outcome in OSCC patients [3,18]. In the present study, in the first phase, we graded all 325 cases with WHO and modified Bryne's grading systems. Not surprisingly, the latter showed strong inter-observer agreement. It should further be noted that in 1992, Bryne's system excluded mitotic count as it did not seem to affect the prognosis; however, we found that retaining the mitotic count in grading improves the agreement between two observers. We believe that as AI reckons on learning appropriate features, it is essential to use uniform grading criteria for the selection and aggregation of valid features and consequent recognition for unwavering results.

Previous studies have employed CNN such as InceptionV3, VGG16, VGG19, and ResNet-50 for the classification of histological subtypes for OSCC images [19]. AlexNet, in particular, has been studied for the prognostication of tumor-infiltrating lymphocytes in non-small cell lung cancers [20]. There are available data regarding the estimation of accuracy utilizing oral submucous fibrosis and OSCC images. Cumulatively, we found accuracy ranging from 64.31% to 89.2% for all three neural networks; however, individually, the accuracy was variable. The same held true for the other parameters analyzed. The accuracy for WDSCC cases by InceptionV3 was 68.3%, which was much lower than SqueezeNet (75.8%) and VGG16 (73.8%). The highest accuracy was noted for the classification of PDSCC (grade 3 OSCC) using all three neural networks; it is noteworthy that uni- and multivariate Cox regression analyses and the log-rank test of 85 OSCC cases showed poor disease-specific survival of grade 3 tumors [3]. Panigrahi et al., in a comparative evaluation, found higher accuracy of 96.6% using ResNet-50 (precision value of 97% and recall value of 96%) as compared to VGG16, VGG19, InceptionV3, and MobileNet [21]. The accuracy in previous studies has shown variegated data which could be attributed to different grading systems used warranting the application of a universal grading system bearing a definitive prognostic value. Uniformity in the grading system with procurement of images based on specific parameters would result in precise domains.

AUC depicts the diagnostic accuracy of quantitative tests. In cases when the receiver operating characteristic (ROC) corresponds to a random chance, AUC equals 0.5, while a value of 1.0 was considered as perfect accuracy. The probabilities for assigning random positive examples over random negative examples were 0.790, 0.748, and 0.715 using InceptionV3, SqueezeNet, and VGG16 for MDSCC. Excellent discrimination was noted for WDSCC and PDSCC for all three networks (score of >0.8). Ananthakrishnan et al. used a dual approach for the analysis of images for differentiation between normal and neoplastic tissues. Their proposed approach extracted the features related to the images using pre-trained CNN and further trained a classification model using the resulting feature vectors. An accuracy of 96.94% and an AUC of 0.976 were found for images at 400X magnification [22]. In the present study, we evaluated 325 OSCC cases, and images were taken at 100X magnification with an AUC of >0.8; interestingly, PDSCC images showed the highest AUC of more than 0.9 using all three CNNs using the Orange data mining tool, which is a simple, free open-source image analyzing tool for image analysis, with some inherent limitations. Utilizing the Keras Python library, the Orange data mining tool provides an interface to several DL models for image analytics [13]. These values were comparable with other reported data. 

Limitations

The present study has few limitations. Firstly, the sample size was not uniform among the three groups (based on availability), and the mining tools provide better precision with more images and adequate training. Secondly, only one field, with most poorly differentiated grade, was selected for each case, which could be a source of confounding error. Future studies including whole slide imaging may help in overcoming these issues. Thirdly, as the study included images of consecutive recent cases, inclusion of long-term follow-up data was not possible. However, the results provide a baseline data for future studies.

## Conclusions

AI is a novel approach which could serve as adjunct for improvement in theragnostics. It is however prudent to believe that DL-based learning requires further research targeting the understanding of the different digital methods and validation of different available tools to achieve not only greater PVs, sensitivity, specificity, AUC, and accuracy but also better security. Uniform guidelines for the selection of images and universal grading system for OSCC with a definitive prognostic value (Bryne's) are warranted for the better application of AI in image analysis of OSCC images. 
